# Family Income Gradients in Adolescent Obesity, Overweight and Adiposity Persist in Extremely Deprived and Extremely Affluent Neighbourhoods but Not in Middle-Class Neighbourhoods: Evidence from the UK Millennium Cohort Study

**DOI:** 10.3390/ijerph17020418

**Published:** 2020-01-08

**Authors:** Michael Osei Mireku, Alina Rodriguez

**Affiliations:** 1 School of Psychology, University of Lincoln, Lincoln LN6 7TS, UK; 2 Department of Epidemiology and Biostatistics, School of Public Health, Imperial College London, London W2 1PG, UK; a.rodriguez@imperial.ac.uk

**Keywords:** socioeconomic, deprivation, geographic variation, adolescence, obesity, adiposity, BMI, inequality, family income

## Abstract

We investigated whether family income gradients in obesity, overweight, and adiposity persist at geographic-level deprivation quintiles using a nationally representative cohort of UK adolescents. Data from 11,714 eligible adolescents from the sixth sweep of the Millennium Cohort Study (14 years old) were analysed in this study. The International Obesity Task Force age- and sex-specific thresholds were used to define obesity and overweight. Self-reported family income was standardized using the Organisation for Economic Co-operation and Development (OECD)’s equivalised income scale. Geographic-level deprivation was defined by the index of multiple deprivation 2004. Results showed that the prevalence of obesity and overweight was 8.0% and 27.2%, respectively. Mean percentage body fat was 16.9% (standard error, *SE* = 0.2%) in male and 27.3% (*SE* = 0.1%) in female adolescents. Risk of obesity, overweight, and adiposity increased with decreasing family income quintiles (*p* for trend <0.001). After stratifying by geographic-level deprivation quintiles, a U-shaped association emerged, whereby family income gradients in the risk of adolescent obesity and adiposity persisted in extremely affluent and extremely deprived neighbourhoods but attenuated to non-significance in middle-class neighbourhoods. These results focus on the findings from England. Recognition of the persistence of inequalities in the risk of obesity in the most deprived and affluent neighbourhoods may be necessary in planning public health resources and interventions.

## 1. Introduction

Globally, there has been a dramatic increase in the prevalence of obesity [1]. The most problematic upsurge in obesity prevalence has been among children and adolescents—for whom, there has been a reported 1.2 kg/m^2^ increase in global age-standardised average body mass index (BMI) since 1975 to 2016 [2]. Over this same period, the global prevalence of obesity has increased by eight- and nine-folds for girls and boys, respectively [2]. According to the WHO, there are 41 million children between 5 and 16 years who are classified as being overweight or obese [3]. However, in the recent decade, evidence from the UK suggests that there has been a gradual tapering in adolescent obesity prevalence, particularly for children from wealthy families but not for those from poorer families [4].

Obesity in childhood is a major public health burden and a concern for policy makers because of the associated adverse health outcomes during childhood and across the lifespan [5]. Obese and overweight children are more likely to have social and emotional problems in adolescence mainly due to low self-esteem and exclusion by peers [6] as well as neurodevelopmental problems [7]. Obesity in children is likely to persist in adulthood [8]. A recent meta-analysis of published literature based on data gathered in high-income countries revealed that obese and overweight children were at risk of high systolic and diastolic blood pressure, high total cholesterol and triglycerides and higher insulin resistance in childhood [9]. These intermediate risk factors are indicators of potential metabolic syndrome later in life and could be markers of early onset of cardiovascular disease and type-II diabetes in adulthood [10,11].

At the individual-level, family income and socioeconomic inequalities are well-established risk factors for childhood obesity and overweight [12,13,14,15]. Indeed, socioeconomic position has an additive impact in predicting weight beyond genetic liability [16]. These studies show decreasing family income and socioeconomic indicators are related to increasing risk of childhood obesity and overweight. Likewise, studies looking at socioeconomic deprivation at the geographic level also report similar gradients in risk of obesity and overweight [17]. In contrast, in low- and middle-income countries (LMICs), affluence is positively related to obesity [18]. Although recent literature suggests the prevalence of childhood obesity in the UK within the past decade is levelling-off, albeit high, there is substantive evidence to suggest that this levelling-off is absent in areas with the highest geographic-level deprivation [4,19]. Geographic-level deprivation in this paper is defined as the relative level of impoverishment of a neighbourhood or a geographic area measured by infrastructure, health services, job opportunities, crime, etc. We hypothesised that family income gradients in the risk of adolescent obesity, overweight and adiposity will be evident independent of the level of geographic deprivation. In the present study, we investigated whether family income gradients in obesity, overweight, and adiposity persist for every geographic-level deprivation quintile using a nationally representative cohort of UK adolescents.

## 2. Materials and Methods

### 2.1. Data Sources

The UK Millennium Cohort Study (MCS) is an ongoing nationally representative prospective cohort study of live born children between September 2000 and January 2002. Using a stratified, clustered random sample design, the MCS disproportionately over-sampled hard-to-reach populations including ethnic minorities and those living in smaller countries of the UK (i.e., Wales, Northern Ireland, and Scotland) and in disadvantaged areas. Sampling and attrition weights were generated to account for the sub-group oversampling in the design and attrition at each sweep. Details of the sampling strategy and profile of the MCS have been explained elsewhere [20]. Briefly, eligible children were selected from universal child benefit records in the UK when they were 9 months old. However, children of asylum-seeking parents were ineligible [20]. The present study uses data from the sixth sweep of MCS (MCS6), *N* = 11,726, which represents the mid-adolescent (aged approximately 14 years) follow-up [21]. We restricted our analysis to the first registered child of any participating family thus every participating family was represented by a single child. 

### 2.2. Measures

#### 2.2.1. Anthropometric and Body Fat Measures

Anthropometric and body fat measures of children were taken by qualified investigators. Height (to the nearest 0.1 cm) was measured using a Leicester Height Measure Stadiometers (Seca Ltd., Birmingham, UK). Children’s weight (to the nearest 0.1 kg) and percentage of body fat were taken using electronic Tanita™ scales (Tanita UK Ltd., Middlesex, UK). Body mass index (BMI) was calculated by dividing the weight (in kg) by the square of the height (in m). Of the 11,714 children with eligible survey data (i.e., 12 did not have survey characteristics), BMI was only available for (93.5%) and body fat percentage available for (92.4%). The International Obesity Task Force (IOTF) age- and sex-specific thresholds were used to define obesity and overweight [22]. 

#### 2.2.2. Family Income

Equivalised family income was obtained by dividing self-reported total net weekly household income by the number of household members according their assigned weight (i.e., 1 for first adult, 0.5 for each remaining adult, and 0.3 for each child under 14 years) on the Organisation for Economic Co-operation and Development (OECD)’s equivalised income scale. The equivalised income is an indicator for household disposable income. Quintiles of equivalised income were generated by the MCS team and used in our analyses. The highest income quintile was used as the reference. 

#### 2.2.3. Geographic-Level Deprivation

Geographic-level deprivation was defined by the overall index of multiple deprivation (IMD) 2004. The overall IMD was generated from combining seven domain indices of neighbourhood deprivation: local authority geographic-level crime, living environment deprivation, income deprivation, health deprivation and disability, education skills and training deprivation, employment deprivation, and barriers housing and services. These indices were generated at the level of geographical units, developed by the UK Office for National Statistics, called Lower Layer Super Output Areas (LSOAs). Scores assigned to each of the 32,482 LSOAs in England were ranked for the IMD [23]. Deciles of the overall IMD (~3248 LSOAs in each) generated by the MCS team were collapsed into quintiles of geographic-level deprivation in our analyses. Because there is no uniformity in the definition of geographic-level deprivation across countries, the overall IMD were only available for country-specific analysis and not UK-wide analysis. 

#### 2.2.4. Covariates

The following potential confounding variables were considered for the analysis: child’s age (in years), sex at birth, ethnicity, highest level of mother’s education, physical activity, and sedentary activity. Maternal education was obtained from parents’ questionnaire whiles physical and sedentary activities were self-reported by adolescents. The sedentary activity variable was derived from maternal reports of hours of the day spent watching TV or videos from the computer. Maternal education variable was defined by the level of National Vocational Qualification. Age was used as a continuous variable. The remaining confounding variables were collapsed into binary variables (e.g., ethnicity—white/non-white; mother’s highest educational level—below higher education certificate/higher education certificate or higher, etc.) as shown in Table 1. These confounding variables were dichotomised to reduce potential empty categories arising from the several levels of stratification. 

### 2.3. Statistical Analysis

Log-binomial generalised linear models were run to assess the relative risk (RR) of obesity and overweight among each of the four lower family income quintile categories compared to highest income quintile category. The reference category for the dependent variables, obesity and overweight, were non-obese and non-overweight, respectively. The adjusted linearity of the family income gradient on adiposity and the risk of obesity and overweight was assessed. To examine the association between equivalised family income quintiles and percentage body fat, we used linear regression models for survey data. Highest income quintile was used as the reference income quintile in all analyses. Mean differences (MD) in percentage body fat between each of the four lower family income quintiles and the highest income quintile were calculated in multiple linear regression models adjusted for child’s age, sex, ethnicity, physical activity, sedentary activity, and highest level of mother’s education. All the above models were run for the UK-wide dataset and country-specific datasets using the appropriate survey weighting. 

Country-specific analyses were restricted to only the England sub-cohort (*N* = 7724) because multiple regression analyses involving stratification by geographic-level deprivation quintiles resulted in zero sub-population in one or more strata or non-convergence in the Scotland, Wales and Northern Ireland datasets. Linear regression models and log-binomial models for the association between family income quintiles and adiposity, obesity and overweight prevalence were run for each quintile of geographic-level deprivation for the England sub-cohort. Linear trends in family income gradient on each of the outcomes were assessed for each geographic-level deprivation quintile. 

All statistical analyses were conducted by using Stata SE/15.1 (Stata Corp, College Station, TX). All statistical analysis accounted for survey weights. Significance was defined as *p* < 0.05 and regression models results were reported MD or RR with 95% confidence intervals (CI). 

### 2.4. Ethical Consideration

Ethical approval for MCS6, including subsequent amendments, was obtained from National Research Ethics Service (NRES) Research Ethics Committee London by CLS (REC ref: 13/LO/1786) [24]. The MCS dataset is publicly available on the UK Data Service website to registered users. The present project received governance approval from the University of Lincoln Research Ethics Committee (ref: 2019-1093).

## 3. Results

Sociodemographic characteristics of the study population by equivalised family income quintiles are displayed in Table 1. Except for sex, all sociodemographic different variables varied by family.

Income quintiles and linearly increased or decreased from the lowest family income quintile to the highest family income quintile (*p* for trend <0.001). Participants in the lowest income quintile category had the largest proportion of non-white ethnicity, lower physical activity, higher sedentary activity and mothers with lower educational level, compared to each of the remaining four higher income quintiles. The prevalence of obesity was 8% in the entire UK cohort and this was consistent across the four countries of the UK (England, 8%; Northern Ireland, 8%; Scotland, 8%; Wales, 10%). In all, 27% of UK adolescents were overweight. The prevalence of adolescent obesity and overweight, and percentage body fat increased from the highest income quintile to the lowest income quintile (*p* for trend <0.001). 

Table 2 summarises the characteristics of the England sub-cohort by quintiles of geographic-level deprivation. 

Among adolescents in the England sub-cohort, the prevalence of obesity and overweight increased with increasing level of geographic-level deprivation: 4.4% and 21.1% (least deprived quintile) to 7.6% and 26.6% (third deprived quintile) to 10.7% and 32.5% (most deprived quintile), respectively. Likewise, percentage of body fat increased with increasing level of geographic deprivation (*p* for trend <0.001). There was a clear gradient in socioeconomic and sociodemographic characteristics by geographic-level deprivation quintiles. For example, the proportion educated mothers and physically active adolescents decreased with increasing level of geographic deprivation (Table 2). Of those in the richest quintile, 45.2% lived in least deprived quintile communities while only 1.8% of this group lived in the most deprived quintile communities. Contrarily, 46.5% of the poorest quintile families lived in the most deprived quintile communities and only 2.9% of them lived in the least deprived quintile communities (Table 2).

Table 3 shows the crude and adjusted RRs and MDs for the relationships between the risk of obesity, overweight, adiposity, and equivalised family income for the entire UK cohort and the England sub-cohort. In this section, references are made to only adjusted models. In the entire UK cohort, adolescents in the third family income quintile had 1.2% (95% CI: 0.7–1.8) more body fat whereas those in the lowest income quintile had 2.7% (95% CI: 1.8–3.6) more body fat compared to highest income quintile (Table 3). Similarly, family income gradients were visible in the prevalence of adolescent obesity and overweight (*p* for trend <0.001). Adolescents in the lowest family income quintile had 2.4 (95% CI: 1.7–3.4) times higher risk of being obese compared to those in the highest income quintile (Table 3). 

As shown in Table 3, the effect sizes reported in the entire UK cohort were consistent with those reported in the England sub-cohort. Family income gradients in the risk of adolescent obesity, overweight and adiposity were all statistically significant (*p* for trend <0.001). 

**Figure 1:**
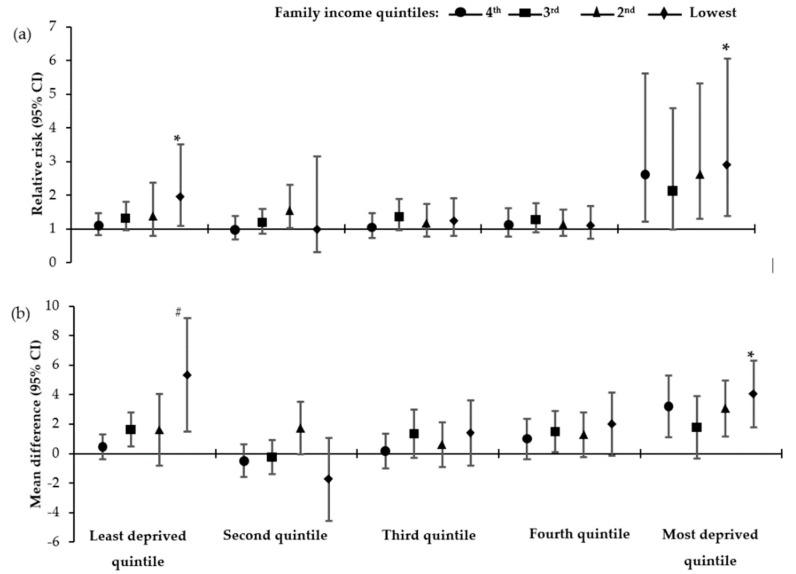
Association between overweight/obesity risk and adiposity and equivalised family income quintiles stratified by quintiles of geographic-level deprivation. (**a**) Relative risk (95% CI) of overweight/obesity by family income quintiles—model adjusted for sex, age, ethnicity, maternal education, physical activity, sedentary activity; (**b**) Mean difference (95% CI) of percentage body fat by family income quintiles—model adjusted for sex, ethnicity, maternal education, physical activity, sedentary activity. Reference category is the highest family income quintile. * *p* for trend<0.05; ^#^ *p* for trend <0.01. Unless otherwise stated, *p* for trend for the model was >0.05.

After stratifying the adjusted models by quintiles of geographic-level deprivation for the England sub-cohort, the family income gradient in the risk of adolescent obesity, previously observed in Table 3, attenuated to non-significance for those who resided in neighbourhoods that were classified as the second, third or fourth quintile of deprivation. Further, in these neighbourhoods, the risk of obesity for any of the poorest family income quintiles compared to the highest family income quintile was not statistically significant. On the contrary, family income gradient in the risk of adolescent obesity persisted for the least and most deprived quintiles of geographic-level deprivation (Table 4). Similarly, as shown in Figure 1, family income gradients in the risk of overweight and percentage body fat among adolescents were visible for those living in the most deprived quintile neighbourhoods and those living in the least deprived quintile neighbourhoods.

## 4. Discussion

In this large and contemporary cohort of UK adolescents, we found linear trends in increasing risk of obesity, overweight and adiposity with decreasing level of family income and increasing level of geographic-level deprivation, independently. After stratification by geographic-level deprivation, there was no significant linear trend in the association between family income quintiles and the risk of obesity, overweight, or percentage body fat for adolescents living in moderately affluent neighbourhoods (second to fourth geographic-level deprivation quintiles). However, family income gradients in the risk of obesity, overweight and adiposity persisted in neighbourhoods in the top and bottom quintiles of geographic-level deprivation. 

The present study adds to the growing body of literature highlighting socioeconomic inequalities in paediatric health. In this cohort, there was 140% increased risk of obesity for adolescents from families in the lowest socioeconomic (family income) quintile compared to those in the highest socioeconomic quintile, and the risk of obesity and overweight increased with decreasing individual-level socioeconomic status. Similar trends in socioeconomic inequalities in the risk of childhood and adolescent obesity have been reported in other large studies [14,25]. Although at the individual-level, the aetiology of obesity may be simply explained by energy imbalance i.e., increased energy intake and reduced energy expenditure, the pathways to inequality in childhood and adolescent obesity are rather complicated [26]. Using structural equation modelling, a recent study in eight cities in Europe revealed that two potential indirect pathways between parental socioeconomic status and childhood obesity: via access to open green space and physical activity, and solely via physical activity [12]. Most studies consistently report that the inverse socioeconomic-level trends in the risk of obesity and overweight is mostly explained by inequalities in parent-level variables other than at the child level [4,25,27]. In fact, a recent simulation analysis on the MCS data revealed that even if all children achieved the World Health Organisation’s physical activity recommendations, the relative socioeconomic gradient in obesity and overweight risk will persist even though the prevalence of obesity will massively decrease [28]. 

In this study, equivalised family income was chosen as the indicator for individual-level socioeconomic status because it provided information about the combined status of the household and represents the buying power for families, including income that is used for food purchases. That said, studies that used highest level of maternal and/or paternal academic qualifications as indicators of socioeconomic status have found similar socioeconomic gradients in the risk of child obesity and overweight [13,29,30]. Several studies that used neighbourhood characteristics as indicators for socioeconomic class have reported inequalities in the risk of adolescent obesity and overweight at the geographic level, which is consistent with our findings in the England subpopulation [31,32,33]. The prevalence of obesity and overweight linearly increased from 4% and 21% among the adolescents living in communities in the least deprived quintile to 11% and 33% among those living the most deprived communities. Although several recent studies show virtual stability or moderate declines in the trends of childhood obesity in England, the gains appear to disproportionately benefit wealthy people and wealthy communities than the poorest ones [4,19,34]. 

In the present study, we investigated whether family income gradients in the risk of obesity, overweight, and adiposity persist in each quintile of geographic-level deprivation after stratification. Family income gradients disappeared for the second to fourth geographic-level deprivation quintiles but persisted for adolescents living in the most affluent quintile and most deprived quintile communities. The persistent family income gradients in the risks of adolescent obesity, overweight, and adiposity in the extreme quintiles of geographic deprivation may be explained by individual-level inequality gaps within the extremes of geographic-level deprivation. As mentioned earlier, the geographic-level deprivation index is a composite of factors such as accessibility to health facilities, jobs, healthcare, housing, etc. Also, there are several LSOAs within a town or city, implying that an LSOA in the richest 20% of LSOAs could border an LSOA in the poorest 20% of LSOAs. Thus, it is likely that individuals in the richest income quintiles who live the poorest neighbourhoods, can access amenities and favourable environments in richer neighbourhoods. Similarly, many amenities in the wealthiest neighbourhoods may be private or the extent of accessibility increases with increasing income. On the contrary, ease of access to community resources in moderately affluent diminishes the increasing advantage of individual-level wealth on the risk of childhood obesity, overweight and adiposity. Although our study is not the first to investigate the relationship between geographic-level deprivation, individual-level socio economic status and adolescent obesity we found very few studies that have considered these factors contemporaneously. A similar study in Sweden found that increasing neighbourhood-level deprivation to be independently associated with increasing risk of childhood obesity [35]. However, this study did not stratify analysis by geographic-level deprivation. 

Obesity in childhood is a serious public health problem. Several studies investigating obesity/BMI tracking suggests that it occurs early in childhood and continues through adolescence into adulthood [36,37,38]. Recent literature suggest that childhood obesity onsets at 2 years with the most rapid weight gain occurring by age 6 years [38]. Thus, children who are obese at 6 years are more likely to remain obese in adolescence and subsequently, in adulthood. Our study sample included adolescents who may or may not have undergone puberty. The onset of puberty is earlier among the poorest compared to the richest children [39]. These socioeconomic differences in the onset of puberty may explain the family income gradients in the risk of obesity and overweight in the study sample. However, we could not further stratify our analysis by sex to account for the menarche as this resulted in in zero sub-population in one or more strata or non-convergence.

Reducing inequalities in childhood obesity has social and economic benefits and therefore has warranted political nation-wide or targeted interventions in many developed countries. In the UK, it is estimated £2 billion is spent on treating diseases that are associated with socioeconomic inequalities [40]. Several recent government policies and interventions including levy on soft drinks, and 20% sugar reduction in processed foods have been implemented to reduce childhood obesity [40]. However, our findings suggest that additional targeted interventions in the least and most deprived communities may reap benefits in reducing the family income gradients in the risk of obesity. 

Our study has several strengths. We used data from a nationally representative sample to investigate the family income gradients in the risks of obesity, overweight and adiposity for different levels of geographic-level deprivation. The large sample of our data permitted the use of quintiles of socioeconomic and further stratification of our analysis by quintiles of geographic-level deprivation. However, this was only true for the England sub-cohort not the other three countries of the UK for which the sample sizes were not large enough. In addition, the lack of UK-wide geographic level deprivation index did not allow us to investigate our principal hypothesis across the entire UK population. Another strength of our study is the consistent findings across all measures i.e., obesity, overweight and percentage body fat. Even though BMI is sufficient in discriminating obese and non-obese at a population level, it is widely known that percentage body fat is a better predictor of chronic disease. Hence, the inclusion of percentage body fat data further strengthens the findings. That said, BMI and body fat measures were not available for all adolescents—about 8% of the study sample did not have data on body fat. 

## 5. Conclusions

Family income gradient in adolescent obesity and adiposity persists in extremely affluent and extremely deprived neighbourhoods but the gradient becomes blurred in middle-class neighbourhoods. Although our results confirm that poorer families and poorer neighbourhoods have increased risk of adolescent obesity compared to richer families or neighbourhoods, they demonstrate that decreasing individual-level socioeconomic status is not universally associated with increasing adolescent obesity risk. Reducing inequality gaps in the most deprived and the most affluent neighbourhoods in the UK may be advisable. 

## Figures and Tables

**Table 1:** Characteristics of study population by equivalised family income quintiles (*N* = 11,714).

Characteristics	Equivalised Family Income Quintiles					
	Highest	Fourth	Third	Second	Lowest	*p* for Trend
Sex						
Boys	51.6	54.0	51.9	51.1	53.4	0.467
Girls	48.4	46.0	48.1	48.9	46.6	
Ethnicity ^1^						
White	90.7	88.3	84.4	78.7	60.5	<0.001
Non-white	9.3	11.7	15.6	21.3	39.5	
Mother’s highest educational level ^1^						
<HE certificate	23.4	50.3	61.9	75.7	89.6	<0.001
≥HE certificate	76.6	49.7	38.1	24.3	10.4	
Moderate–vigorous PA ^1^						
<3 days per week	21.7	27.1	32.4	30.6	34.3	<0.001
≥3 days per week	78.3	72.9	67.6	69.4	65.6	
TV/video watching^1^						
≥3 h per day	36.1	43.8	45.5	47.5	48.6	<0.001
<3 h per day	63.9	56.2	54.5	52.5	51.4	
Obesity^1^						
Obese	3.7	5.8	8.3	10.4	12.3	<0.001
Not obese	96.3	94.2	91.7	89.7	87.7	
Overweight ^1^						
Overweight/obese	19.5	23.8	29.1	30.1	34.1	<0.001
Not overweight/obese	80.5	76.2	70.9	69.9	65.9	
Percentage body fat	20.1 (0.2)	21.0 (0.2)	22.2 (0.2)	22.7 (0.3)	23.3 (0.4)	<0.001

HE—higher education; PA—physical activity. The International Obesity Task Force age- and sex-specific thresholds were used to define obesity and overweight. ^1^ Missing data for PA (*N* = 346), TV/video watching (*N* = 338), ethnicity (*N* = 449), mother’s education (*N* = 1471), obesity/overweight (*N* = 755) and percentage body fat (*N* = 894).

**Table 2:** Characteristics of sub-cohort in England by geographic-level deprivation quintiles (*N* = 7716).

Characteristics	Geographic-Level Deprivation (IMD) Quintiles					
	Least	Second	Third	Fourth	Most	*p* for Trend
Sex						
Boys	53.1	51.4	53.1	53.1	52.1	0.921
Girls	46.9	48.6	46.9	46.9	47.9	
Ethnicity ^1^						
White	88.3	87.2	82.8	76.0	60.5	<0.001
Non-white	11.7	12.8	17.2	24.0	39.5	
Mother’s highest educational level ^1^						
<HE certificate	40.1	45.5	60.6	65.0	72.4	<0.001
≥HE certificate	59.9	54.5	39.4	35.0	27.6	
Moderate–vigorous PA ^1^						
<3 days per week	22.2	28.2	31.3	35.7	32.0	<0.001
≥3 days per week	77.8	71.9	68.7	64.3	68.0	
TV/video watching ^1^						
≥3 h per day	36.1	43.8	45.5	47.5	48.6	<0.001
<3 h per day	60.2	58.4	54.3	50.1	53.5	
Equivalised family income quintile						
Highest	45.2	31.3	18.0	9.6	1.8	<0.001
Fourth	25.8	27.9	26.1	18.5	6.1	
Third	17.9	20.9	25.8	21.9	15.4	
Second	8.2	13.1	17.7	26.4	30.2	
Lowest	2.9	6.9	12.5	23.6	46.5	
Obesity ^1^						
Obese	4.4	6.0	7.6	9.8	10.7	<0.001
Not obese	95.6	94.0	92.4	90.2	89.3	
Overweight ^1^						
Overweight/obese	21.1	22.9	26.6	30.3	32.5	<0.001
Not overweight/obese	78.9	77.1	73.5	69.7	67.5	
Percentage body fat	20.1 (0.3)	20.9 (0.3)	21.6 (0.3)	22.5 (0.3)	23.1 (0.4)	<0.001

HE—higher education; PA—physical activity. ^1^ Missing data for PA (*N* = 242), TV/video watching (*N* = 235), ethnicity (*N* = 306), mother’s education (*N* = 1110), obesity/overweight (*N* = 520) and percentage body fat (*N* = 616).

**Table 3:** Risk of obesity, overweight and adiposity by equivalised family income quintiles in the entire UK cohort (A) and in the England sub-cohort (B).

	Obesity		Overweight		Percentage Body Fat	
Income Quintiles	RR (95% CI) ^1^	RR (95% CI) ^3^	RR (95% CI) ^1^	RR (95% CI) ^3^	MD (95% CI) ^2^	MD (95% CI) ^4^
*A. Analysis in entire UK cohort*						
Highest ^5^	1.0	1.0	1.0	1.0	0.0	0.0
Fourth	1.4 (1.0–1.8) *	1.3 (1.0–1.8) *	1.2 (1.1–1.4) ^#^	1.2 (1.0–1.3) *	0.9 (0.4–1.4) ^≠^	0.7 (0.2–1.2) ^#^
Third	1.8 (1.3–2.4) ^≠^	1.7 (1.2–2.3) ^#^	1.4 (1.2–1.6) ^≠^	1.3 (1.1–1.5) ^≠^	1.5 (0.9–2.0) ^≠^	1.2 (0.7–1.8) ^≠^
Second	2.1 (1.5–2.8) ^≠^	1.9 (1.4–2.7) ^≠^	1.5 (1.3–1.7) ^≠^	1.4 (1.2–1.6) ^≠^	2.1 (1.4–2.7) ^≠^	1.8 (1.2–2.4) ^≠^
Lowest	2.7 (1.9–3.7) ^≠^	2.4 (1.7–3.4) ^≠^	1.6 (1.4–1.9) ^≠^	1.5 (1.3–1.8) ^≠^	3.0 (2.2–3.9) ^≠^	2.7 (1.8–3.6) ^≠^
*B. Analysis in England sub-cohort*						
Highest ^5^	1.0	1.0	1.0	1.0	0.0	0.0
Fourth	1.2 (0.9–1.7)	1.1 (0.8–1.6)	1.2 (1.0–1.4) *	1.1 (1.0–1.3)	0.8 (0.3–1.4) ^#^	0.6 (0.1–1.2) *
Third	1.8 (1.3–2.6) ^≠^	1.7 (1.2–2.4) ^#^	1.4 (1.2–1.7) ^≠^	1.4 (1.2–1.6) ^≠^	1.6 (1.0–2.2) ^≠^	1.4 (0.8–2.0) ^≠^
Second	2.1 (1.4–3.0) ^≠^	1.9 (1.3–2.8) ^#^	1.5 (1.3–1.8) ^≠^	1.4 (1.2–1.7) ^≠^	2.2 (1.5–2.9) ^≠^	1.9 (1.2–2.6) ^≠^
Lowest	2.5 (1.7–3.8) ^≠^	2.2 (1.5–3.3) ^≠^	1.6 (1.4–1.9) ^≠^	1.5 (1.3–1.8) ^≠^	3.1 (2.1–4.1) ^≠^	2.7 (1.7–3.7) ^≠^

*p* for trend for all models < 0.001; * *p* < 0.05; ^#^ *p* < 0.01, ^≠^ *p* < 0.001. RR—relative risk; MD—mean difference. ^1^ Model adjusted for age, ethnicity, maternal education; ^2^ Model adjusted for sex, age, ethnicity, maternal education; ^3^ Model adjusted for age, ethnicity, maternal education, physical activity, sedentary activity; ^4^ Model adjusted for sex, age, ethnicity, maternal education, physical activity, sedentary activity. ^5^ Reference—equivalised family income quintile.

**Table 4:** Family income gradient in the risk of obesity, RR (95% CI), by geographic-level deprivation quintiles.

Family Income Quintiles		Geographic-Level Deprivation Quintiles			
	Least	Second	Third	Fourth	Most
(a) Partially adjusted model					
Highest ^1^	1.0	1.0	1.0	1.0	1.0
Fourth	1.4 (0.7–2.8)	0.7 (0.3–1.5)	0.9 (0.4–2.2)	1.6 (0.7–3.9)	3.1 (0.6–17.3)
Third	2.2 (1.1–4.3)	0.7 (0.3–1.6)	2.2 (1.0–4.8) *	2.2 (0.9–5.2)	2.4 (0.4–13.0)
Second	4.3 (1.8–10.4) ^#^	1.4 (0.6–3.1)	1.1 (0.4–3.5)	1.7 (0.8–3.9)	4.1 (0.8–21.1)
Lowest	5.5 (1.8–17.2) *	0.2 (0.0–1.6)	2.7 (1.0–7.2)	2.4 (1.0–5.8) *	4.4 (0.9–22.3)
***p* for trend**	<0.001	0.990	0.052	0.133	0.040
(b) Fully adjusted model					
Highest ^1^	1.0	1.0	1.0	1.0	1.0
Fourth	1.3 (0.6–2.6)	0.7 (0.3–1.5)	0.8 (0.4–1.9)	1.5 (0.6–3.7)	3.1 (0.6–17.3)
Third	1.9 (1.0–3.8)	0.7 (0.3–1.6)	1.9 (0.9–4.2)	2.1 (0.9–4.7)	2.4 (0.4–13.1)
Second	3.9 (1.6–9.4) ^#^	1.5 (0.7–3.2)	1.0 (0.3–2.9)	1.6 (0.7–3.7)	4.1 (0.8–21.3)
Lowest	4.8 (1.4–16.8) *	0.2 (0.0–1.4)	2.1 (0.8–5.4)	2.0 (0.8–5.0)	4.4 (0.9–22.4)
***p* for trend**	<0.001	0.956	0.096	0.336	0.039

* *p* < 0.05; ^#^ *p* < 0.01. RR—relative risk. ^1^ Reference category of equivalised family income quintile; (a) Adjusted for age, ethnicity, and maternal education; (b) Adjusted for age, ethnicity, maternal education, physical activity, and sedentary activity.
